# Mechanical Properties of Epoxy Compounds Based on Bisphenol a Aged in Aqueous Environments

**DOI:** 10.3390/polym13060952

**Published:** 2021-03-19

**Authors:** Anna Rudawska

**Affiliations:** Faculty of Mechanical Engineering, Lublin University of Technology, 20-618 Lublin, Poland; a.rudawska@pollub.pl; Tel.: +48-81-538-4232

**Keywords:** mechanical properties, epoxy compounds, aqueous environments, ageing

## Abstract

(1) Background: The aim of the work is to determine the influence of selected aqueous environments of various types of liquids on the strength of adhesive compositions prepared from epoxy resin based on bisphenol A combined with two different curing agents: tritethylenetetramine and polyaminoamide C. (2) Methods: The cured epoxy adhesive compounds samples were seasoned in four aqueous environments of the liquid: rainwater, demineralized water, tap water, and a sweetened drink. Three variants of the aging time in the above-mentioned operating environments were adopted: one month, two months, and three months. After the specified maturing time, samples of epoxy adhesive compositions were subjected to the strength tests on the Zwick/Roell 150 testing machine, which is in accordance with ISO 604 standard, determining the compressive strength. (3) Results: On the basis of the obtained strength test results and their analysis, it was noticed, inter alia, that the strength of the epoxy compounds decreases with the aging time in all used aqueous environments. Moreover, in the case of both types of the epoxy compounds, the highest strength was achieved after aging in demineralized water.

## 1. Introduction

A significant advantage of epoxy compounds as adhesive is the possibility of joining together most of the available materials (in any combinations and arrangements), such as glass, metal, wood, ceramics, polymer materials, etc. due to their excellent adhesion. There is no second method that allows for so many possibilities of joining the different materials. Another advantage of epoxy compounds is good mechanical properties and resistance to many chemical factors, and the adhesive layer is characterized by excellent dielectric properties and allows the exclusion of moisture from entering the joint [1,2,3,4].

The operating conditions of epoxy compounds may have a large impact on their strength, as well as the strength of adhesive joints made with the use of various epoxy compounds [2,5,6,7,8]. One of the important factors determining the strength and durability of the epoxy compounds is the natural environment of use, which includes a water environment with different water content and in various forms [8,9,10,11,12]. On the other hand, the epoxy resins are not as susceptible to degradation under the influence of water compared to other resins due to the lack of hydrolysis sensitive ester groups in their molecular structure. This issue is considered in many publications [13,14,15,16,17,18]. Several of the author’s works also deal with the issue of aging various epoxy compounds in various environments [19,20,21,22], including acid [23] and also salt [24] environments. Based on obtained results by Rudawska and Brunella [25], it has been found that too high iron sulfate content in water has a negative effect on the selected mechanical properties of epoxy adhesive compound. The de-ionized water, salt, and sea water were used as aqueous aging environments in the studied presented by Bordes et al. [7]. In this work, the long-term behavior of adhesively bonded steel joints aged in mentioned environments and the degradation of mechanical properties of an epoxy adhesive were considered. According to Gao et al. [26], shear strength tests indicated that the durability of the epoxy adhesive in salt water aging is lower than in water. In salt water, Cl^−^ and Na^+^ ions can accelerate hydrolization of the epoxy adhesive and enhance water ingress ability in the epoxy adhesive. This statement is also emphasized by Wang et al. [27].

Under conditions of high humidity, epoxy compounds may change their strength reversibly or irreversibly, which was presented and confirmed in many works [8,10,12,13]. De Neve and Shanahan [10] considered the aging of an epoxy adhesive based on DGEBA in water vapor (*ca*. 100% RH) at various elevated temperatures. Based on the results, it can be noticed that long-term exposure of the epoxy adhesive to water leads to both physical and chemical degradation of the material. Fernades et al. [8] presented the fracture envelope of a commercial epoxy adhesive as a function of the water content in the epoxy adhesive. The distilled water and salt water were used as aging environments. The obtained results presented that the toughness of the epoxy adhesives changed as a function of the type of ageing environment. On the one hand, the mechanical properties increased for the salt water environment; on the other hand, for the distilled water environment, degradation of the mechanical properties of epoxy adhesives was observed. The results presented by Yang et al. [12] indicated that exposure to moisture results in plasticization and decreases in the performance characteristics of epoxy adhesives. Lettieri and Frigione [13] examined the effects of exposure to different humid environments in a commercial epoxy resin. Plasticization, reactivation of curing reactions, and erasure of physical ageing were observed in the specimens subjected to the different humidity regimes and all affecting both the thermal and the mechanical properties of the aged samples. 

In order to prevent the epoxy compounds from weakening the strength of the epoxy compounds, this problem should be anticipated at the beginning of the formulation.

The issues analyzed in the study concern selected operating factors and their impact on the strength of epoxy compound. The subject of this study is to compare the effect of operating factors on the strength of epoxy compounds made of epoxy resin based on bisphenol A and two different curing agents: tritethylenetetramine and polyaminoamide C. A comparative analysis of the operating factors of the epoxy compounds will be carried out in terms of the compressive strength. This is to determine which operating environment is more favorable for the strength results obtained in the epoxy compounds.

## 2. Materials and Methods

### 2.1. Epoxy Compounds

Two types of the epoxy compounds were used in the experimental tests, containing the epoxy resin made of bisphenol A (Epidian 57 modified epoxy resin—trade name, producer: CIECH Resins, Nowa Sarzyna, Poland) as the basic component and two types of curing agent: triethylentetraamine (TECZA, Z-1—trade name, producer: CIECH Resins, Nowa Sarzyna, Poland), and polyaminoamide C (PAC—trade name, producer: CIECH Resins, Nowa Sarzyna, Poland). The epoxy resin used is a synthetic resin made of epichlorohydrin (ECH) and dian (bisphenol A, BPA, (4,4’-isopropylidenediphenol). The epoxy number of the used epoxy resin amounts to 0.40 mol/100 g. This resin belongs to the group of low molecular weight resins. The prepared epoxy compounds are presented in Table 1.

Epidian 57 epoxy resin is a mixture of Epidian 5 epoxy resin (unmodified epoxy resin based on bisphenol A, epoxy number amounts 0.49–0.52 mol/100 g) with modified polyester resin [28]. It is a homogeneous and viscous liquid. The epoxy adhesive containing this type of epoxy resin is used for “cold” bonding of glass, metals, thermosetting polymers, ceramics, leather, and rubber. Epidian 57 is a medium-elastic epoxy resin with good tear resistance and high shear strength. As a result of curing process, this epoxy resin loses its transparency, taking on the color and appearance of ivory [28]. To use Epidian 57 epoxy resin as adhesive (adhesive compound), a suitable curing agent should be used. The choice of the epoxy compounds containing Epidian 57 epoxy resin for testing was related to the fact that this resin is very often used as the basic component of epoxy adhesives intended for joining many construction materials.

The triethylenetetramine (TECZA) and polyaminoamide C curing agents were used to cure the epoxy compounds. The selected properties of the curing agents were presented in Table 2.

**Figure 1:**
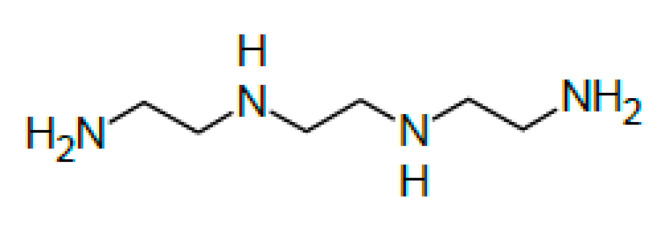
Structural formula of triethylenetetramine curing agent (Triethylenetetramine. Available online: https://pl.qaz.wiki/wiki/Triethylenetetramine (accessed on 9 March 2021)).

The triethylenetetramine (TECZA, Z-1 trade name) is used for curing reactive resins [19]. The chemical formula of the triethylenetetramine curing agent is C_6_H_18_N_4_, and the structural formula is shown in Figure 1.

It is most often used with low molecular weight (liquid) epoxy resins or in various compounds based on them. The spatial cross-linking of epoxy resins occurs as a result of chemical reactions of functional groups contained in the resin (epoxy and hydroxide) with a properly selected curing agent. The curing process takes place when the curing agent is added to the resin and the gelling time begins from that moment. After the gelling period, the resin is quickly cured. It is important to remember that the use of the triethylenetetramine curing agent requires precise dosing and weighing, as any excess curing agent will shorten its gelation time. Incorrect weight proportions also result in deterioration of the properties of the cured material. The gel time of the triethylenetetramine curing at room temperature is about 35 min. Initial curing is obtained after about 3–4 h, and after 48 h, the degree of curing of the composition is about 80–90%. It takes 7 to 14 days to fully cure. When there is a need to shorten the curing time of a given compound, the triethylenetetramine curing agent can also be used for the curing at an increased temperature [28]. The triethylenetetramine curing agent, in which the functional groups are connected with each other by short aliphatic chains, causes that after the curing process the products are, among others, relatively stiff and brittle at ambient temperature.

Polyaminoamide C curing agent (PAC trade name) is used with the low molecular weight (liquid) epoxy resins and with various compounds based on them. The weight ratio of the polyaminoamide C curing agent to the epoxy resin can be varied within a fairly wide range by controlling the reaction rate and properties of the cured material [20]. The polyaminoamide curing agent is one of the flexability-increasing curing agents that contain relatively few functional groups, whose groups are separated by long aliphatic chains. The epoxy compounds with more curing agents are characterized by their better flexibility and impact resistance. Unfortunately, also for this reason, the resistance to elevated temperature and the hardness of the compounds formed is lower than in the compounds with the predominance of epoxy resin. The polyaminoamide C curing agent is classified as a slow-reacting curing agent. The gel time of the curing agent at room temperature is approximately 180 min. Full curing is achieved after 7–14 days [28]. This is an important parameter in the further processing of these epoxy compounds.

One of these epoxy compounds shows the characteristics of a more rigid material (Epidian 57/Z-1/10:1), while the other composition (Epidian 57/PAC/1:1) shows the characteristics of a more flexible material than the first epoxy compounds.

### 2.2. Shape and Epoxy Compounds Technology

**Figure 2:**
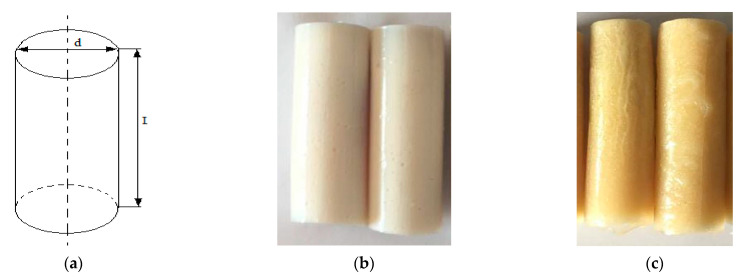
Epoxy compounds samples: (**a**) scheme; (**b**) Epidian 57/Z1/10:1, (**c**) Epidian 57/PAC/1:1.

For the strength tests, the cylinder-shaped samples of the epoxy compounds were used, with the following dimensions: diameter *d* = 20 mm and length *L* = 60 mm (Figure 2).

Proper preparation of the cylinder-shaped molds with dimensions *d* = 20 mm and *L* = 60 mm for the preparation of samples of the epoxy compounds is necessary to separate the cured epoxy compounds from the surface of the molds used after the curing process. In the research, POLSIFORM (Polish Silicones, Nowa Sarzyna, Poland) was used as a silicone anti-adhesive agent, preventing the prepared the epoxy compounds from sticking to the molds. After spraying, the preparation creates a thin layer of silicone oil on the surface of the mold, which has an anti-adhesive effect. Silicone oil does not in any way affect the properties of the prepared epoxy compounds and does not cause some defects in the finished products. Before use, the agent was shaken and then sprayed onto the mold surface from a distance of about 30 cm. Three minutes after the release of the release agent, the mold was filled with the liquid epoxy compound. This process was carried out at a temperature of 22 ± 2 °C, with an air humidity of 21–23%.

Before applying the epoxy compounds to the molds, the selected components should be carefully combined. The combined components of the compound were mixed in the appropriate proportions resulting from the stoichiometric ratio (the quantitative ratio of the reactants in the chemical reaction—molar, weight, or volume) for E57/Z-1/10:1 epoxy compound and above the stoichiometric ratio for E57/PAC/1:1 epoxy compound. 

The mixture of Epidian 57 epoxy resin and polyaminoamide C curing agent was mixed in the ratio of 1: 1, while with the polyaminoamide C curing agent was mixed in the ratio of 10: 1. A TP-2/1 scale (manufacturer FAWAG S.A., Lublin Factory of Scales, Lublin, Poland, ISO9001 certified) was used to measure the ingredients, with a maximum weight of 2000 g and an accuracy of 0.1 g. The components of epoxy compounds were mixed for 90 s with a disk agitator at 128 m/min in a polymer container on the mixing station. The mixing continued for 2 min; then, the epoxy compounds were kept under vacuum for a further 2 min, in order to remove gases and avoid the formation of bubbles. An important element in creating the epoxy compound is to avoid the formation of air bubbles. Air bubbles can significantly reduce the strength of the epoxy compound. The epoxy compound preparation process was done at 22 ± 2 °C and 21–23% RH.

In order for the epoxy compounds to be carefully made, special attention should be paid to the correct application of the compounds to the cylindrical molds, after prior careful preparation of the molds. Then, the epoxy compounds were subjected to a one-stage curing process for 7 days at a room temperature of 22 ± 2 °C and air humidity of 21–23%.

### 2.3. Aging Environments and Conditions

The water (hydrogen oxide) is a compound composed of two hydrogen atoms and one oxygen atom: H_2_O. The water does not exist in nature as a pure hydrogen–oxygen compound. As one of the few substances, it can exist in three states of aggregation: gas, as water vapor, liquid, as rain, and solid, as ice. In each state of aggregation, water has the same chemical composition and similar structure, but its behavior may be different. Water is always a very dilute solution of acids, bases, salts, and gases. The chemical composition of any water depends on its origin. The rainwater contains a small amount of various admixtures. Surface waters are characterized by a high content of substances of industrial and natural origin. The water for the production of the drinking water undergoes various treatment processes. The treatment process depends on the amount and type of contaminants removed from it. There are several compounds in the chemical language that are called water, but they have a completely different composition than real water.

The experimental studies used the three types of water and a sweetened drink. The aqueous environments’ characteristics are presented in Table 3.

The rainwater is formed by the condensation of water vapor that is formed in the atmosphere and falls back to the Earth’s surface in the form of precipitation such as rain, snow, or hail. The composition of the rainwater depends on the purity of the air. The rainwater is characterized by a large amount of gases (nitrogen, oxygen, and carbon dioxide). It may also contain the microorganisms, the industrial dust, the soot, and small amounts of the mineral salts. The pH content in rainwater is about 6, which is acidic due to the content of dissolved carbon dioxide. The demineralized water is the water devoid of mineral salts and most of the other pollutants by repeated distillation. It contains dissolved gases (oxygen, carbon dioxide, and nitrogen. The tap water, otherwise known as drinking water, is clean water that can be consumed without endangering human health and life. It contains the right amount of the mineral salts and does not contain the inorganic and the organic impurities. The used sweetened drink is a sweet carbonated drink that was initially made from the coca bush and the fruit juice of the kola tree, mixed with soda water.

The cured samples of the epoxy compounds were subjected to the aging process in the various aqueous environments described in Table 4.

**Figure 3:**
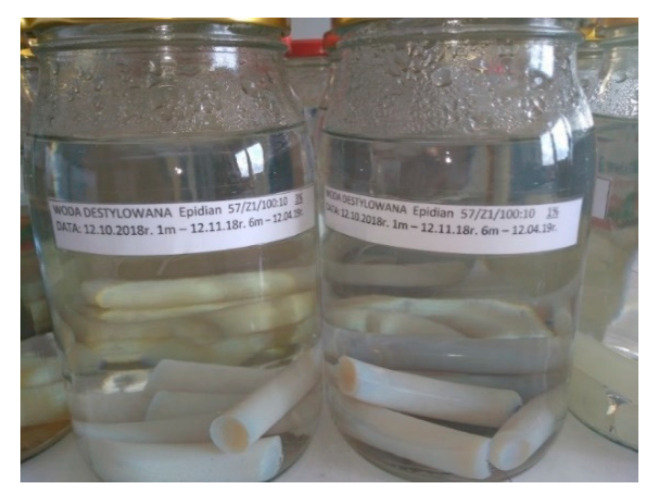
The examples of two glass vessels with E57/Z-1/10:1 epoxy compound samples immersed in rainwater and tap water.

The aqueous environments were placed in the glass vessels. The samples of the epoxy compounds were immersed in the aqueous environments (Table 3) in such a way that the aqueous solutions fully covered the samples, and then, the glass vessels were tightly closed. The examples of two glass vessels with E57/Z-1/10:1 epoxy compound samples immersed in the rainwater and the tap water are shown in Figure 3.

For each aging variant, 5 samples of two types of the epoxy compounds were prepared. A total of 120 samples of the epoxy compounds were made (5 samples × 2 epoxy compounds × 3 seasoning variants × 4 operating conditions).

### 2.4. Test

Tests of the cured epoxy compounds were carried out in accordance with ISO 604 on a test stand consisting of a Zwick/Roell Z150 testing machine (ZwickRoell GmbH&Co. KG, Ulm, Germany) and a computer set connected to a testing machine. The samples were successively subjected to the strength tests at predetermined dates.

**Figure 4:**
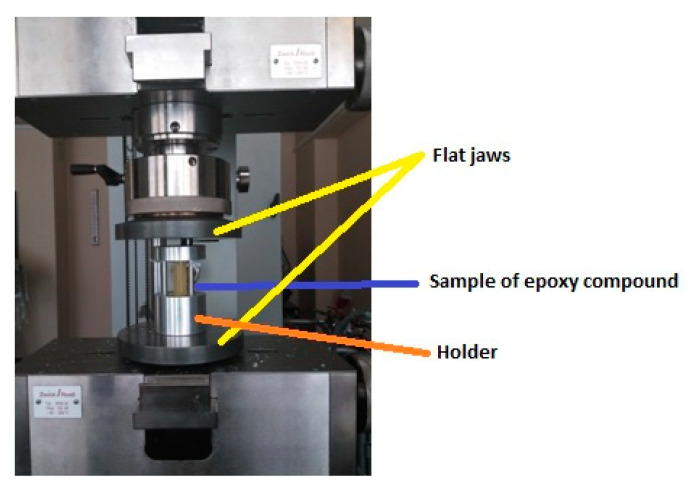
View of the mounted epoxy compound sample.

The samples of the epoxy compounds were placed in a special holder, which in turn was placed in the flat jaws of the testing machine. Mounting the samples in an additional holder allowed maintaining the coaxiality of the sample in relation to the axis of the traverse and the action of the axial force as well as perpendicularity to the surface of the holders of the testing machine. The holder with mounted cylindrical samples of the epoxy compounds placed in the flat jaws of the testing machine is presented in Figure 4.

The strength tests concerned the determination of the compressive strength, taking into account four different variants of the aqueous environments: rainwater, demineralized water, tap water, and sweetened drink. During the strength test, the initial force was 10 N, the traverse speed was 10 mm/min, and the maximum deformation was set at 15%.

## 3. Results

### 3.1. Strength Test—Epidian 57/Z-1/10:1 Epoxy Compound

**Figure 5:**
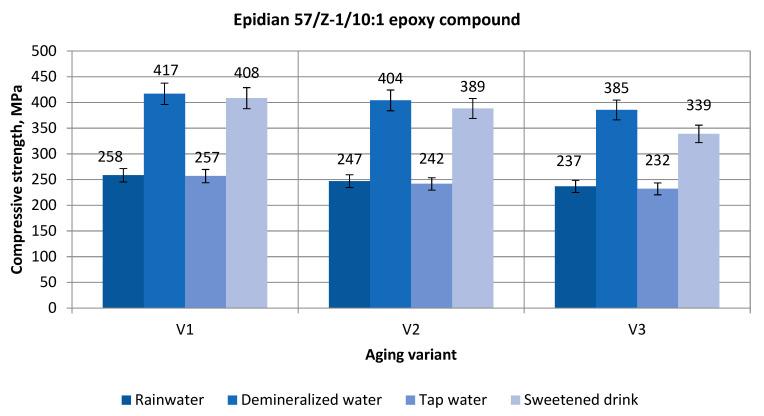
Compressive strength of Epidian 57/Z-1/10:1 epoxy compound samples aging in various aqueous environments.

Figure 5 shows the effect of aging on the strength of the Epidian 57/Z-1/10:1 epoxy compounds samples, aged in three aging variants in four aqueous environments.

Based on a comparison of the compressive strength test results (Figure 2), it was observed that:

- In all variants of aging, the highest compressive strength was achieved after aging in the demineralized water, and the lowest was achieved after aging in the tap water;
- Increasing the aging time reduces the value of the compressive strength of the samples of Epidian 57/Z-1/10 1 epoxy compound;
- The percentage decrease after 2 months of sample aging in the rainwater is 4.4%; for the demineralized water, it is 3%; for the tap water, it is 5.9%; and it is 4.9% for the sweetened drink;
- The percentage decrease after 3 months of the sample aging in the rainwater is 4.2%, while it is 4.7% for the demineralized water, 4% for the tap water, and 12.7% for the sweetened drink.

Comparing the results of the research in relation to the aging variant I (1 month), the following can be noted:

- The highest maximum strength was achieved after aging in demineralized water; it amounts to 412 MPa (V1-DW);
- The lowest compressive strength of the epoxy compound in the cured state was achieved after aging in the tap water, amounting to 257 MPa, which is 62% of the highest value of the strength achieved after aging in the demineralized water;
- The compressive strength after aging in the rainwater is 62% of the highest compressive strength (258 MPa);
- After aging in the sweetened drink (in V1-SD), the average value of the compressive strength was achieved at the level of 408 MPa, which is 98% of the strength of the epoxy compounds aged in demineralized water.

When analyzing the test results for the 2nd variant of aging (2 months), it was noticed that:

- The highest strength achieved after aging in the demineralized water (V2-DW) is 404 MPa;
- The lowest compressive strength of the epoxy compound in the cured state was achieved after aging in tap water (V2-TW) (242 MPa), which is 60% of the highest strength value (404 MPa);
- The compressive strength after aging in rainwater (V2-RW) is 39% lower than the highest compressive strength;
- After aging in the sweetened drink (V2-SD), the compressive strength is lower by 4% compared with the compounds aged in the demineralized water.

When considering the obtained test results in the case of the 3rd variant of aging (V3-3 months), it was noticed that:

- The highest maximum strength was achieved after aging in the demineralized water (V3-DW), and it amounts to 385 MPa, and the lowest compressive strength of the epoxy compounds in the hardened state was achieved after aging in the tap water (V3-TW), amounting to 232 MPa, which is 60.2% of the highest strength value;
- After aging in the rainwater (V3-RW), the compressive strength (237 MPa) is nearly 40% lower than the highest strength;
- After aging in the sweetened drink (V3-SD), the average value of the compressive strength was achieved at the level of 339 MPa, which is 88.0% of the strength of the epoxy compounds aged in the demineralized water.

### 3.2. Strength Test—Epidian 57/PAC/1:1 Epoxy Compound

**Figure 6:**
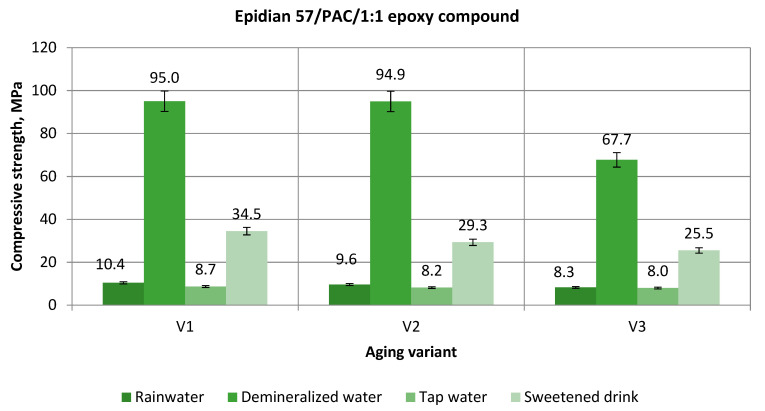
Compressive strength of Epidian 57/PAC/1:1 epoxy compound samples aged in various aqueous environments.

Figure 6 shows the effect of aging on the strength of the Epidian 57/PAC/1:1 epoxy compounds samples, which were aged in three aging variants in four aqueous environments.

Considering the obtained results of the compressive strength of Epidian 57/PAC/1:1 epoxy compounds, presented in Figure 6, the following can be noted:

- The strength of the considered epoxy compounds decreases with the increase of the aging time in various environments;
- The epoxy compounds aged in the demineralized water achieved the highest strength at any time of aging;
- The lowest strength is characteristic of the epoxy compounds immersed in the tap water, at any time of aging;
- The percentage decrease after 2 months of the epoxy compound samples aging in the rainwater is 7.7%; for the demineralized water, it is 0.11%; for the tap water, it is 5.7%; and for the sweetened drink, it is 15.1%;
- The percentage decrease after 3 months of sample aging in the rainwater is 13.5%, for the demineralized water, it is 2.4%, for the tap water, it is 28.7%, and for the sweetened drink, it is 13%.

Comparing the results of the research in relation to the aging variant I (V1), the following can be noted:

- The highest maximum strength was achieved after aging in the demineralized water, and it amounts to 95.0 MPa. On the other hand, the lowest compressive strength of the epoxy compound samples in the hardened state was achieved after seasoning in the tap water, amounting to 8.7 MPa, which is 9.2% of the highest value of strength achieved in the demineralized water;
- The compressive strength of aging in the rainwater is 10.9% of the highest compressive strength;
- After aging in the sweetened drink, the average value of the compressive strength was achieved at the level of 34.5 MPa, which is 36.3% of the strength of the epoxy compounds aged in the demineralized water.

When analyzing the obtained values of the compressive strength for the 2nd variant of aging during 2 months (V2), the following can be noted:

- The highest maximum strength was achieved after aging in the demineralized water and it amounts to 94.9 MPa, and the lowest was achieved after aging in the tap water, which was 8.2 MPa, and it constitutes 8.6% of the highest strength value;
- After aging in the rainwater for two months (V2-RW), the compressive strength is 90% lower than the compressive strength after aging in the demineralized water (V2-DW);
- The second variant of the aging in the sweetened drink (V2-SD) allowed achieving the compressive strength of 29.3 MPa, which is 30.9% of the strength of the epoxy compounds aged in the demineralized water (V2-DW).

The compressive strength for the 3rd aging variant (V3) was as follows:

- The compressive strength after aging for three months reached the highest value after aging in the demineralized water (V3-DW)—67.7 MPa, and the lowest was achieved in tap water, amounting to 8.0 MPa (V3-TW), which is 11.8% of the highest strength value;
- After aging in rainwater (V3-RW), the compressive strength is 12.3% of the highest compressive strength;
- After aging in the sweetened drink (V3-SD), the average value of the compressive strength was achieved at the level of 25.5 MPa, which constitutes 37.7% of the strength of the epoxy compounds seasoned in the demineralized water (V3-DW).

### 3.3. Visual Analysis—Epidian 57/Z-1/10:1 Epoxy Compounds

The images of Epidian 57/Z-1/10:1 epoxy compounds samples after the strength tests (taken with a digital camera) are presented in Table 5, Table 6 and Table 7.

The visual analysis of the samples of the epoxy compounds subjected to various aging variants (Table 4) was made on the basis of the external appearance, shape, and color. With regard to the external appearance and shape, it was noticed that along with the increase in the aging duration, the deformation of the epoxy compound samples increases. The length of samples is shortened, and there is a deformation at the ends of the samples (especially in the case of variant I, see Table 5). There are only some minor scratches on the samples circumference. In the case of aging within 2 months (Table 6) of the epoxy compounds, scratches and cracks also appear. There are some signs of shape deformation, especially buckling. At the longest analyzed aging variant (variant III, Table 7), a cohesive failure of the epoxy compounds samples was noticed, with a clear appearance of some cracks and also the other material defects: spalling. The fracture of these samples for variant III is typical for the materials showing the features of the brittle materials. The visual analysis of the pictures revealed that the kind of damage experienced by Epidian 57/Z-1/10:1 epoxy compound specimens mainly depends on the aging duration, while it apparently was not affected by three aqueous environments: the rainwater, the demineralized water, and the tap water. However, aging can be seen in the sweetened drink.

It was noticed that in the case of water, the color changed very little with regard to the different types of water used. On the other hand, the use of aging in the sweetened drink caused a significant change in the color of the samples. Initially, the samples had a milky white color, while after aging in the sweetened drink, the samples turned brown. The intensity of this color increased with the aging time in this solution. The epoxy compound samples aged in the sweetened drink also show a similar nature of damage for variant I and variant II. On the other hand, aging for 3 months in the sweetened drink (V3-SD) seems to have less influence on the cohesive failure of the samples than in the case of other water environments (Table 6). In this case (V3-SD), less destructive failure was observed than in the remaining samples, and no spalling of the samples was observed. Perhaps the ingredients of the sweetened drink help to reduce the brittleness of these epoxy compounds.

### 3.4. Visual Analysis—Epidian 57/PAC/1:1 Epoxy Compounds

The Epidian 57/PAC/1:1 epoxy compounds samples after the strength tests are presented in Table 8, Table 9 and Table 10.

When analyzing the shape and appearance of the Epidian 57PAC/1:1 epoxy compound samples after the strength tests, minor scratches with no distinct signs of samples deformation were observed (Table 8 and Table 9). There are a lot of scratches—a network of minor cracks—on the samples circumference ((Table 10) of the Epidian 57PAC/1:1 epoxy compound samples after 3 months of aging (Variant III). The samples of the Epidian 57PAC/1:1 adhesive compound after strength tests show a tendency to scratches with increasing aging time.

There is a significant difference in the color of the samples when comparing samples aged in the three types of the water and in a sweetened drink. The epoxy compound samples aged in a sweetened drink take on a brown color, which is likely due to the type of aging time in the sweetened drink. Color intensity increases with increasing aging time. The darkest brown color occurred in the samples aged for 3 months (V3-SD). From the photos of aging in the sweetened drink in Table 8, Table 9 and Table 10, it can also be seen that the volume of the samples in which the color has changed increases with increasing aging time. The color change in the entire sample volume is visible in the case of variant III (V3-SD). On the other hand, the color change only at the edge of the samples (outer layer of samples) is visible in samples V1-SD and slightly greater for samples V2-SD.

On the other hand, no significant differences in the type of deformation of the samples of epoxy compounds subjected to the aging in the sweetened drink were observed, when comparing individual variants of aging. There is also a larger mesh of cracks during the 3 months of seasoning in a sweetened drink as well as when aging in different types of water for 3 months. However, cracks and delamination at the ends of the epoxy compound samples can be noticed.

By observing the results, it is possible to notice a different nature of the damage of the Epidian 57ZPAC/1:1 epoxy compounds than the samples made of the Epidian 57Z-1/10:1 epoxy compounds. The samples of the Epidian 57Z-1/10:1 epoxy compound are more prone to deformation, which is revealed by the test shapes buckling and spalling during compression.

Differences were also noticed in the susceptibility to color change under the influence of dyes in the sweet drink. A significant color change was noticed in the case of the Epidian 57/PAC/1:1 epoxy compounds, with the intensity of the color (up to dark brown) increasing along with the aging time.

From this, it can be assumed that the type of curing agent in the epoxy compounds contributes to the compound being more or less given a color change. This can be aesthetically important when making certain articles of this epoxy compounds (e.g., coatings).

## 4. Discussion

There are significant differences in the values of the compressive strength of the analyzed epoxy compound depending on (i) the type of epoxy composition—the type of curing agent included in the epoxy compounds composition, containing the same epoxy resin, (ii) the type of the environment, and (iii) the aging time.

- Okba et al. [29] underline that the decrease of e.g., residual compressive strengths depends on the type of adhesive and also other factors e.g., elevated temperature level and to a lesser effect on the exposure time. The results presented in work [30] demonstrated that the tensile strength is influenced by both the type of resin and the type of the curing agent; in addition, their ratios in epoxy compounds have an impact on the tensile strength of the specimens in the cured state. The curing agent content has a significant effect on the properties of the epoxy compound even within the range of stoichiometric ratios. In work presented [21], the samples of epoxy compounds with addition of the Z-1 curing agent show a tendency to crack and spall among all tested epoxy compounds, containing different types of curing agent. This tendency was also confirmed in the conducted research;
- Degradation is a process of structural changes that may be the result of physical or chemical changes occurring in polymer materials under the influence of long-term external factors. Most often, these interactions are synergistic and result in interactions between individual stimuli. The aqueous environment (mainly water) was the degradation factor in the work. Most of the materials subjected to long-term exposure to the aquatic environment show a change in some properties. The reduction of mechanical parameters, surface changes observed macroscopically and microscopically, as well as chemical changes are the result of the interaction of hydronium (H_3_O +) and hydroxyl (OH-) ions on the material. Polymer degradation processes are most often associated with structural changes in the polymer chain (and thus with a reduction in molecular weight). This is related to changes in the physicochemical properties of polymers (e.g., solution viscosity, mechanical strength, flexibility, state of aggregation, melting point, solubility). It was found that the samples subjected to aging in dematerialized water, i.e., water free of foreign ions, showed higher strength in the case of both type of epoxy compounds. It can be seen that in the analyzed case, differences in the strength values of epoxy compounds are noticeable. Tap water and rainwater have different ions, and in addition, rainwater may have additional impurities that may weaken the structure of the epoxy cross-linked material;
- Polymer materials react with water according to two mechanisms: mechanical intake and discharge of water and as a result of chemical reactions, e.g., hydrolysis or the formation of • OH and • OH_2_ radicals in the presence of radiation. The epoxy resin is in the form of a long molecular chain with sites reactive at both ends with epoxy groups. The absence of ester groups makes epoxies very water resistant. The epoxy molecule also has two ring groups in the center, which withstand mechanical and thermal stresses much better than linear groups, thanks to which epoxies have very good strength, stiffness, and thermal properties. Due to the fact that their molecular structure does not contain ester groups sensitive to hydrolysis, epoxy resins are less susceptible to water degradation than other types of resins having ester groups. At the same time, remember that all resins retain some moisture. Although, of course, the very intake and discharge of water accelerates the degradation process of polymer materials, in the case of epoxy materials, it is a slower process. The results of the effect of seasoning in water solution containing iron sulfate and the time of seasoning were presented by Rudawska and Brunella [25]. In the study, the influence of the type of water environment on the mechanical properties of epoxy compounds was noticed: lower iron content in water has a more significant impact on increasing the epoxy compound compressive strength than a higher iron content or lack thereof. Rudawska and Frigione [31] showed that the aging regimes affected to a great extent the mechanical properties of modified epoxy compounds. The longer the aging time, the greater the effects on the compressive mechanical properties were observed. On the other hand, the kind of environmental water in each aging regime, i.e., demineralized, distilled, and spring water had a minimal effect. The subjects of the presented research were unmodified epoxy compounds with any fillers and the results are slightly different. It can be seen that another important factor influencing the properties of epoxy compounds is the modification of the epoxy compounds. Another study [23] presented that the increase in the pH of the acidic solution contributed to the decrease in mechanical properties, although the immersion time in the acidic solution is important;
- Rudawska and Frigione [31] showed that the aging regimes affected to a great extent the mechanical properties of modified epoxy compounds. The longer the aging time, the greater the effects on the compressive mechanical properties were observed. On the other hand, the kind of environmental water in each aging regime, i.e., demineralized, distilled, and spring water, had a minimal effect. Based on the results presented by De Neve and Shanahan [10], it can be noticed that long-term exposure of the epoxy adhesive to water contributes to both physical and chemical degradation of the epoxy adhesive. Lettieri and Frigione [14] examined the effects of exposure to different humid environments in a commercial epoxy resin. Plasticization, reactivation of the curing reactions, and erasure of the physical aging were observed in the specimens subjected to the different humidity regimes and all affecting both the thermal and the mechanical properties of the aged epoxy compound samples. On the basis of results presented in [25], it can be noticed that the compressive strength of the epoxy compounds increases with increasing the aging time. The aging time was also considered as one of the operating factors during the aging test, in other works by the authors [19,21,23,31]. It can be observed that both the aging time in combination with other factors, such as temperature or the type of environment, affect the mechanical properties of the epoxy compounds, although the influence is diverse. This time should be considered when analyzing the selection of materials for specific operating environments.

It can be noticed that in many studies, various issues related to the influence of aging in various environments on the strength of epoxy compounds are considered and investigated. Both unmodified compositions and those modified with various fillers are tested. The research methodology is also different; therefore, not all research results are consistent in all aspects with the results presented in the literature. However, the trends or the differences in outcomes are worth noting, which may contribute to a more complete interpretation of the research or design of the experiment.

## 5. Conclusions

The experimental tests concerned the compressive strength of the epoxy compounds in the cured state of the appropriate shape of the samples, where the aging time in various aqueous environments was an important factor.

Based on the results of the strength tests and their analysis, the following conclusions can be drawn:

- The compressive strength of the epoxy compounds decreases with the aging time in all used aqueous environments;
- The more flexible epoxy compound (Epidian 57/PAC/1:1) is characterized by lower compressive strength than the more rigid epoxy compound (Epidian 57/Z1/10:1);
- In the case of both types of prepared epoxy compounds, the highest strength was achieved after aging in the demineralized water, and the lowest was achieved after aging in the tap water, and this is independent of the type of aqueous environment. Therefore, it can be assumed that the content of various types of substances in the tap water adversely affects the mechanical properties of epoxy materials (here, in the form of cured epoxy compound);
- It can also be assumed that the rainwater (similarly to tap water) containing various substances also adversely affects the reduction of the compressive strength of the epoxy compounds compared to the strength of the epoxy compounds aged in the demineralized water, free of many substances and impurities;
- The results of the strength tests obtained after aging in the sweetened drink seem interesting, because this exploitation factor allows for obtaining better strength results than tap water or rainwater. Perhaps the chemical composition of this substance (e.g., various chemical compounds such as bisphenol A, benzene, mercury, phosphoric acid, and others) contributes to such results, although the color should be mentioned after aging the samples of epoxy compound in the sweetened drink, which took a very unsightly appearance, obtaining a brown color (the base color is milk white—Epidian 57/Z-1/10:1 or milky-yellowish of Epidian 57/PAC/1:1).

In conclusion, it can be assumed that the strength of epoxy compounds is significantly influenced by both the type of epoxy compounds, the aging time, and the type of environment in which the compounds will be used. The knowledge of the factors influencing the strength of epoxy compounds allows creating the epoxy compound in such a way that they are characterized by the highest possible strength in selected operating environments. Based on the results obtained, further and extended testing of the epoxy compounds is planned.

## Figures and Tables

**Table 1:** Epoxy compounds and their designation.

Epoxy Resin	Curing Agent	Resin/Curing Agent Ratio	Resin/Curing Agent Amount	Compound Designation
Epidian 57 (epoxy resin made of Bisfenol A)	Triethylentetraamine (Z-1)	100:10	100 g and 10 g	E57/Z-1/10:1
	Polyamide (PAC)	100:100	100 g and 100 g	E57/PAC/1:1

**Table 2:** Selected properties of curing agents.

Selected Properties	Curing Agent	
	Triethylenetetramine	Polyaminoamide C
Amino number [mgKOH/g]	min. 1100	290–360
Density, 20 °C [g/cm^3^]	0.98	1.10–1.20
Viscosity, 25 °C [mPa·s]	20–30	10000–25000
pH value	13.2 in 50% water solution	12.3
Solidification temperature	<−20 °C	3–4 °C
Solubility	dissolves in water dissolves in ketones, esters, alcohols and aromatic hydrocarbons	dissolves in water (771 mg/L) dissolves in acetone

**Table 3:** Characteristics of the aqueous environments.

Aqueous Environment	Characeristics
Rainwater	The pH content in the rainwater is about 6, which is acidic due to the content of dissolved carbon dioxide (Lublin, Poland).
Demineralized water	The demineralized water is free of foreign ions, and the pH of the water used was about 6.5. (Dragon brand, Lublin, Poland).
Tap water	The water is bicarbonate–calcium–magnesium water. It is also the hard water, as the content of calcium carbonates amounts to 381 mg/L (340–510 mg CaCO_3_/L: hard water) [25]. pH 7 is neutral (Lublin, Poland).
Sweetened drink	The ingredients of a sweetened drink are water, sugar, carbon dioxide, color, phosphoric acid, and natural flavors, including caffeine and others. pH is about 2.5.

**Table 4:** Characteristics of the aqueous environments.

Aging Variant	Aging Time	Aqueous Environment		Designation
Variant I	1 month	Rainwater	RW	V1-RW
		Deminetalized water	DW	V1-DW
		Tap water	TW	V1-TW
		Sweetened drink	SD	V1-SD
Variant II	2 months	Rainwater	RW	V2-RW
		Deminetalized water	DW	V2-DW
		Tap water	TW	V2-TW
		Sweetened drink	SD	V2-SD
Variant III	3 months	Rainwater	RW	V3-RW
		Deminetalized water	DW	V3-DW
		Tap water	TW	V3-TW
		Sweetened drink	SD	V3-SD

**Table 5:** View of the shape of the Epidian 57/Z-1/10:1 epoxy compound samples—variant I.

Aging Variants	View of Samples after Strength Test
V1-RW	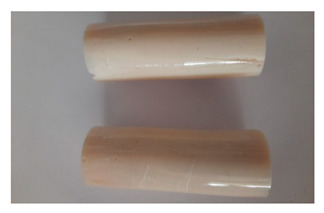
V1-DW	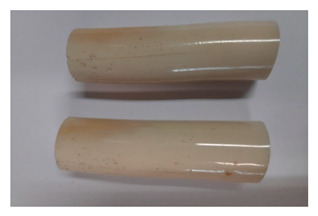
V1-TW	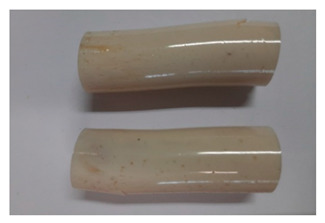
V1-SD	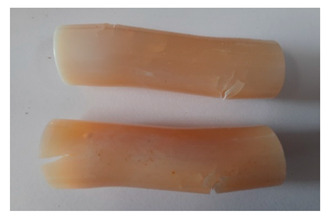

**Table 6:** View of the shape of the Epidian 57/Z-1/10:1 epoxy compounds sample—variant II.

Aging Variants	View of Samples after Strength Test
V2-RW	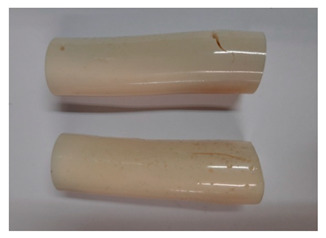
V2-DW	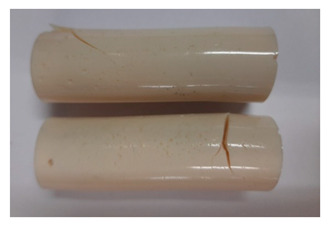
V2-TW	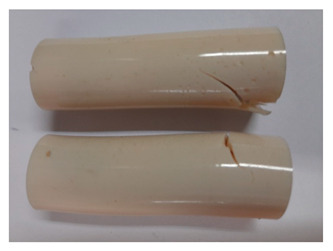
V2-SD	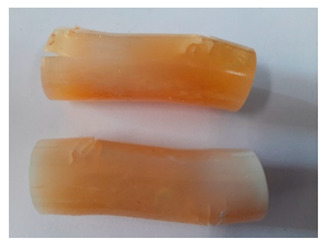

**Table 7:** View of the shape of the Epidian 57/Z-1/10:1 epoxy compounds sample—variant III.

Aging Variants	View of Samples after Strength Test
V3-RW	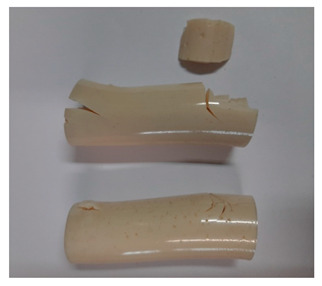
V3-DW	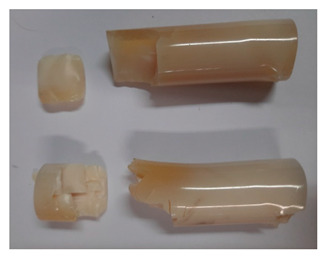
V3-TW	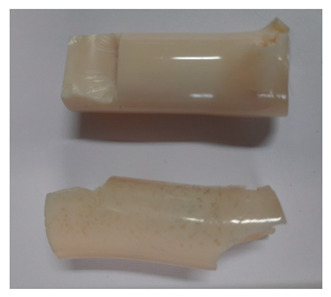
V3-SD	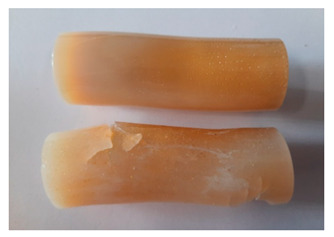

**Table 8:** View of the shape of the Epidian 57/PAC/1:1 epoxy compound samples—variant I.

Aging Variants	View of Samples after Strength Test
V1-RW	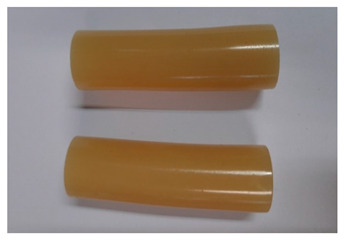
V1-DW	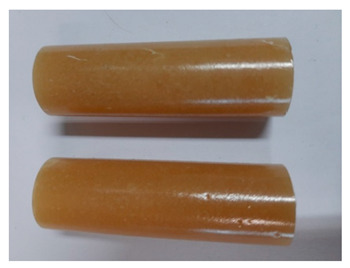
V1-TW	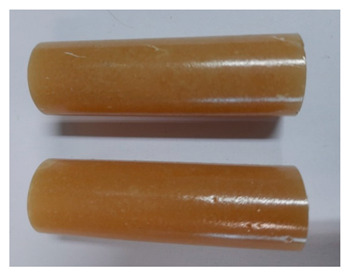
V1-SD	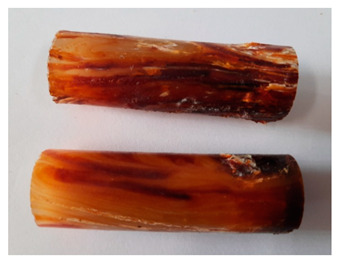
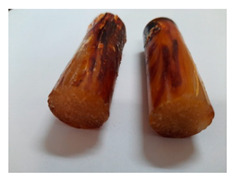	

**Table 9:** View of the shape of the Epidian 57/PAC/10:1 epoxy compound samples—variant II.

Aging Variants	View of Samples after Strength Test
V2-RW	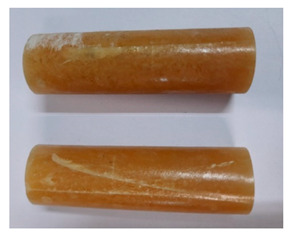
V2-DW	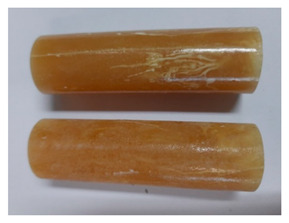
V2-TW	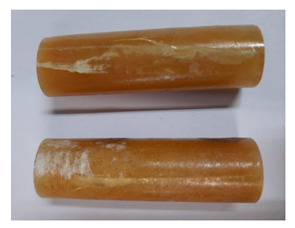
V2-SD	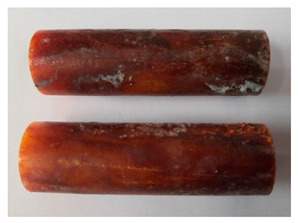
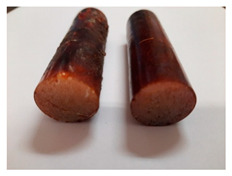	

**Table 10:** View of the shape of the Epidian 57PAC/10:1 epoxy compound samples—variant III.

Aging Variants	View of Samples after Strength Test
V3-RW	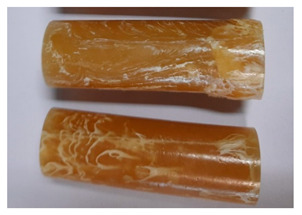
V3-DW	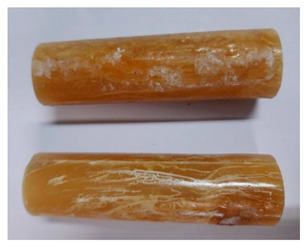
V3-TW	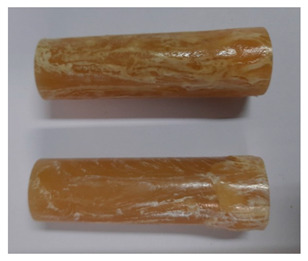
V3-SD	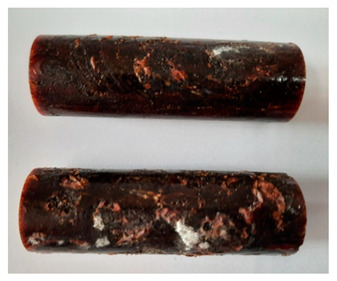
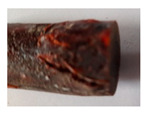	
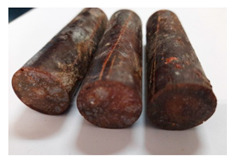	

## Data Availability

The data presented in this study are available on request from the corresponding author.
